# Exploring associations between multidimensional frailty and oral health in community‐dwelling older people. A pilot study

**DOI:** 10.1111/scd.12691

**Published:** 2022-01-10

**Authors:** Erik M. van der Heijden, Wim J. Klüter, Claar D. van der Maarel‐Wierink, Robbert J.J. Gobbens

**Affiliations:** ^1^ Dental Practice Poswick and Van der Heijden Hilversum Netherlands; ^2^ BENECOMO Flemish‐Netherlands Geriatric Oral Research Group Nijmegen Netherlands; ^3^ College of Dental Sciences Radboud University Medical Center Nijmegen Netherlands; ^4^ Department of Oral Medicine Academic Center for Dentistry Amsterdam University of Amsterdam and VU University Amsterdam Netherlands; ^5^ Faculty of Health Sports and Social Work Inholland University of Applied Sciences Amsterdam Netherlands; ^6^ Zonnehuisgroep Amstelland Amstelveen Netherlands; ^7^ Department of Primary and Interdisciplinary Care Faculty of Medicine and Health Sciences University of Antwerp Antwerp Belgium

**Keywords:** community‐dwelling older persons, frailty, multidimensional, oral health, self‐report

## Abstract

**Objective:**

To determine the associations between four validated multidimensional self‐report frailty scales and nine indices of oral health in community‐dwelling older persons.

**Materials and Methods:**

This pilot study was conducted in a sample of 208 older persons aged 70 years and older who visited two dental practices in the Netherlands. Frailty status was measured by four different self‐report frailty questionnaires: Tilburg Frailty Indicator (TFI), Groningen Frailty Indicator (GFI), Sunfrail Checklist (SC), and the Sherbrooke Postal Questionnaire (SPQ). Oral health was assessed by two calibrated examiners.

**Results:**

The prevalence of frailty according to the four frailty measures TFI, GFI, SC, and SPQ was 32.8%, 31.5%, 24.5%, and 49.7%, respectively. The SC correlated with four oral health variables (DMFT, number of teeth, percentage of occlusal contacts, Plaque Index), the TFI with three (number of teeth, percentage of occlusal contacts, Plaque Index), the GFI only with DPSI, and the SPQ with the number of teeth and the number of occlusal contacts.

**Conclusion:**

Of the studied multidimensional frailty scales, the SC and TFI were correlated with most oral health variables (four and three, respectively). However, it should be noticed that these correlations were small.

**Clinical relevance:**

The SC and TFI might help to identify older people with risk of poor oral health so that preventive care can be used to ensure deterioration of oral health and maintenance of quality of life. Vice versa early detection of frailty by oral care professionals could contribute to interprofessional management of frailty.

## 1 INTRODUCTION

Frailty is often regarded as “the opposite of successful aging” and considered to be a better predictor of health outcomes than age itself.^1^ However, frailty is ambiguously defined in the literature. In the absence of a gold standard, there are two approaches to conceptualize and study frailty – either as an unidimensional or a multidimensional concept. The unidimensional concept is characterized by a focus on the physical limitations (unintentional weight loss, fatigue, weakness, slow walking speed, and low physical activity) of older people, such as the frequently cited phenotype of frailty developed by Fried et al.^2^ In contrast, the multidimensional concept of frailty considers several domains of human functioning (e.g., physical, psychological, cognitive, and social factors). The Frailty Index (FI), developed by Mitnitski et al. is multidimensional by nature.^3^ The TI is based on the Canadian Study of Health and Aging Cumulative Deficit Model. It concerns an index of age‐related deficits (at least 30) including diseases and items referring to disability. Examples of frailty scales that also have their premise that frailty is a multidimensional concept are: the Tilburg Frailty Indicator (TFI),^4^ Groningen Frailty Indicator (GFI),^5^ Sunfrail Checklist (SC),^6^ and Sherbrooke Postal Questionnaire (SPQ).^7^ These scales differ from the FI in that they rely on self‐reporting.

Oral health problems such as tooth loss, tooth decay, periodontal disease, reduced masticatory function, and even deterioration of oral health in general are widely prevalent in (frail) older people.^8^ This is worrying because poor oral health decreases social wellbeing and negatively effects general health, for example with a strong association with aspiration pneumonia,^9^ cardiovascular disorders,^10^ and high insulin levels in diabetic patients.^11^

Significant as well as non‐significant associations between frailty and oral health were found.^12^, ^13^, ^14^ A very recently published systematic review on the relationship between oral health factors and frailty among people older than 60 years, resulted in twelve oral health factors, which were grouped in four different categories: oral health status deterioration; deterioration of oral motor skills; chewing, swallowing, and saliva disorders; and oral pain.^15^ Frailty was assessed in 40% of the studies by the physical frailty phenotype (defined as patients having three or more of five frailty components from the Cardiovascular Health Study) in 14% by the Kihon Checklist score, in 7% by the GFI, and in 5% by the FRAIL Questionnaire or the 49‐Item FI. The researchers concluded that their findings could contribute to a possible operational definition of a novel frailty phenotype, defined as an age‐related gradual loss of oral function together with a decline in cognitive and physical functions.

The aim of this pilot study, that was designed 3 years before, was to determine the associations between four validated multidimensional self‐report frailty scales and nine indices of oral health. The authors believe that early detection of frailty by oral care professionals could contribute to the prevention of oral health deterioration and, moreover, could help interprofessional management of frailty.

## 2 METHODS

### 2.1 Study sample and data collection

This pilot study was part of a postgraduate programme and was conducted in Hilversum, a medium sized town in the central part of the Netherlands, and in overasselt, a small village in the eastern part of the Netherlands, between September 2018 and December 2019. Two dentists were involved; both followed a standardized protocol.

The participants were all registered as patients at a dental practice and were asked to participate during their regular visit. At that moment they received a letter informing them about the objective of the study. Inclusion criteria were to be a) aged 75 years or older, because frailty is associated with greater age;^16^ b) dentate in at least one jaw, because dentate persons are more at risk for lack of dental care compared to those wearing full dentures. Furthermore, the results of a systematic review and dose‐response meta‐analysis of prospective cohort studies confirmed a positive relationship between tooth loss and mortality (as final stage of severe frailty). It was concluded that tooth loss may be a potential risk marker for all‐cause mortality. However, their association must be further validated through large prospective studies.^17^ Also, Satake et al. and Tanaka et al. found a role for “fewer teeth” or “tooth loss” in developing oral frailty and functional disability.^18^, ^19^

After a year, it was decided to include also people aged 70 to 74 years in order to achieve the desirable number of people (*N* = 200). After the dental examination, participants were asked to fill in the questionnaire. If they had any problems completing the questionnaire, they were helped by the dentist/examiner.

This study was approved by the Medical Research Ethic Committee VU University Medical Centre Amsterdam (2018.067). All participants provided written informed consent.

### 2.2 Measures

#### 2.2.1 Frailty

Frailty was assessed with four different self‐report frailty questionnaires: TFI,^4^ GFI,^5^ SC,^6^ and the SPQ.^7^ All these measures reflect the multidimensional nature of frailty.

#### 2.2.2 Tilburg frailty indicator

The TFI consists of fifteen items to determine frailty. The items are divided into three domains of frailty: physical frailty (eight), psychological frailty (four), and social frailty (three). The score of total frailty (all fifteen items) ranges from 0 to 15, with greater scores referring to more severe frailty. A cut‐off score of five distinguishes between frail and non‐frail subjects. More details about the items and scoring of the TFI have been published elsewhere.^4^ Many studies have shown that the TFI has good psychometric properties (reliability, validity).^20^, ^21^

#### 2.2.3 Groningen frailty indicator

Like the TFI, the GFI also consists of fifteen items to assess frailty, including the physical (nine), social psychological (five) and the cognitive domains (one). The scoring for total frailty ranges from 0 to 15 with higher scores indicating more frailty. For the GFI, the cut‐off score for frailty is a four.^5^ Many studies have shown that the GFI is a reliable and valid instrument to measure frailty.^22^, ^23^

#### 2.2.4 Sunfrail checklist

The SUNFRAIL tool has been developed in the context of the European SUNFRAIL project aimed to improve the detection, prevention, and management of frailty. This questionnaire includes nine items, divided into three domains: physical (five), neuropsychological (one), and social (three). Higher scores are related to more frailty. The cut‐off score for total frailty is three. Two recent studies have demonstrated that the SUNFRAIL tool has good validity to identify frailty among community‐dwelling older people.^6^, ^24^

#### 2.2.5 Sherbrooke postal questionnaire

The SPQ is a questionnaire that contains six items referring to physical (four), cognitive (one), and social (one) frailty. The score ranges from 0 to 6, with higher scores indicating greater frailty. The established cut‐off score of the SPQ is two. The SPQ has shown good psychometric properties for measuring frailty in samples of community‐dwelling older people.^7^, ^25^

#### 2.2.6 Oral health

Oral health was measured by a complete dental status including the WHO Decayed‐Missing‐Filled Teeth index (DMFT). This method has the advantage of being easy to apply, reaches high levels of reproducibility and excludes pre‐cavitation stages from the measurement of caries lesions. Retained roots and presence of different prosthetic devices such as complete denture, partial dentures (fixed or removable, metal frame or acrylic resin base), bridges, crowns and oral implants were recorded.

For measuring and statistic procedures, nine variables were defined. Besides the DMFT, two concerned caries prevalence and were based on cavitated dentine carious lesions. Because some participants had few teeth, the percentage of carious lesions was calculated in order to correct for tooth loss. This relative variable is thus more relevant than the absolute variable. The fifth and sixth variables were the number and percentage of occlusal contacts. The presence or absence of occlusal contacts was scored on four molars, four premolars, and two cuspidates in the upper jaw. If teeth were replaced by acrylic resin‐based dentures they were scored as no contact. Replacements by metal frame‐based dentures were scored similar to natural teeth.^26^ The Dutch Periodontal Screening Index (DPSI)^27^ was measured as well, as it is based on the health of the periodontium and depth of eventual pockets. The worst periodontium of one of the sextants gives the final DPSI‐score, having a maximum range from 0 to 5. The oral hygiene level of natural teeth was assessed using the Plaque Index described by Silness and Löe at a subset of the so‐called “Ramfjörd teeth” (score range 0–3).^28^ In absence of one of these teeth, the corresponding distal neighbor tooth was assessed. Lastly, the hygiene level of complete or partial dentures was assessed using the method of Augsburger and Elahi (score range 0–4).^29^ In all of these variables, a higher score indicates poorer levels of hygiene.

#### 2.2.7 Sociodemographic characteristics

Sociodemographic variables measured were age, gender, marital status, education, and income. Income was measured by asking “Over the past 12 months, have you struggled to get along with your household's income?” See Table 1 for the answer categories belonging to all sociodemographic characteristics of the participants.

**TABLE 1:** Socio‐demographic characteristics, frailty scales, and oral health characteristics of the participants (*N* = 208)

Characteristic	
Categorical	*n* (%)
*Socio‐demographic characteristics*	
Gender, % of women	120 (57.7)
Marital status	
Married or cohabiting	115 (53.3)
Divorced	15 (7.2)
Not married	7 (3.4)
Widowed	71 (34.1)
Education	
None or primary	22 (10.6)
Secondary	128 (61.9)
Higher	57 (27.5)
Income	
No, do not bother	130 (63.1)
No, do not bother, but I have to keep an eye on my expenses	64 (31.1)
Yes, some difficulty	10 (4.8)
Yes, great difficulty	2 (1.0)
*Frailty*	
Tilburg frailty indicator (TFI) (cut‐off point 5)	59 (32.8)
Groningen frailty indicator (GFI) (cut‐off point 4)	62 (31.5)
Sunfrail checklist (SC) (cut‐off point 3)	49 (24.5)
Sherbrooke postal questionnaire (SPQ) (cut‐off point 2)	99 (49.7)
*Chronic diseases*	
Multimorbidity	94 (45.2)
**Continuous**	**mean (SD), range**
Age	78.6 (5.1), 70–94
*Frailty*	
Tilburg frailty indicator (TFI)	3.5 (2.9), 0–13
Groningen frailty indicator (GFI)	2.7 (2.5), 0–13
Sunfrail checklist (SC)	1.6 (1.3), 0–6
Sherbrooke postal questionnaire (SPQ)	1.7 (1.4), 0–6
*Chronic diseases*	
Number of chronic diseases	1.8 (1.9), 0–8
*Oral health*	
DMFT	25.14 (3.98), 11–32
Number of teeth	19.24 (7.08), 0–31
Number of decayed teeth	0.66 (1.53), 0–16
Percentage decayed teeth	4.22 (10.56), 0–100
Number occlusal contacts	7.52 (3.04), 0–10
Percentage occlusal contacts	81.16 (27.62), 0–100
DPSI	3.70 (1.21), 0–5
Plaque index	1.12 (0.77), 0–3
Augsburger index	0.49 (0.39), 0–1.65

### 2.3 Statistical analyses

Descriptive analyses were used to describe the characteristics of the participants. For categorical variables, the numbers (absolute) and percentages (relative) were determined. For continuous variables the means, standard deviations, and ranges were calculated. Then, we compared the scores on the nine oral health variables for non‐frail and frail people using the established cut‐off scores (see Section 2.2) using Student's t‐tests assuming unequal population variances. In addition, correlations between the four frailty measures and nine oral health variables were examined. According to Cohen, the correlation coefficient (Pearson) was considered as small, medium or large with a coefficient of .1, .3, or .5, respectively.^30^

All data were analyzed using IBM SPSS Statistics 22.0 (IBM, Armonk, NY, USA). All p values reported are two‐tailed. A p value <.05 was considered statistically significant.

## 3 RESULTS

### 3.1 Participant characteristics

The sample consisted of 208 participants; 92.4% agreed to participate, with 120 participants in Hilversum and 88 in Overasselt. Roughly half (58%) of the study population was female. The mean age of the participants was 78.6 (SD = 5.1) years. Of the participants 115 (53.3%) were married or cohabiting. The sample comprised 128 people (61.9%) with secondary education as the highest level of education. Notably, only 12 people (5.8%) experienced some or great difficulty related to their income. Among 45.2% of the participants multimorbidity was present. According to the four frailty scales, the prevalence of frailty using the TFI, GFI, SC, and the SPQ, was 32.8%, 31.5%, 24.5%, and 49.7%, respectively.

In total, 134 participants were dentate in the upper jaw and 155 in the lower. Three participants had complete upper dentures, one had a complete lower denture, and one was edentate in the lower jaw. There were 44 participants with removable partial dentures in the upper jaw, and 50 in the lower jaw.

Regarding cavities, 130 had none, 51 had one, 19 had two, and eight had more than two cavities. Thirteen participants had one tooth‐root present, four had two tooth‐roots, and two had more than two tooth‐roots. There were three participants with one oral implant; three with two oral implants; two with two; and two with more than four oral implants. The socio‐demographic, frailty and oral health characteristics of the participants are presented in Table 1.

### 3.2 Differences between groups on oral health variables and multimorbidity

Table 2 shows differences in the scores on oral health variables of frail and non‐frail participants assessed with TFI, SC, and SPQ. Frail participants assessed with the SC and the SPQ had lower number of teeth and number of occlusal contacts, while they scored higher on the Plaque Index. Moreover, participants who were frail according the TFI scored lower on percentage of occlusal contacts. The oral health variables DMFT, percentage and number decayed teeth and Augsburger Index did not demonstrate significant differences between the two groups. In addition, frailty measured with the GFI did not provide a statistical difference between frail and non‐frail participants with regard to any of the nine oral health variables. Therefore, these results are not shown in Table 2.

**TABLE 2:** Comparison of the scores on oral health variables between non‐frail and frail participants

	TFI				SC			
	Non‐frail	Frail			Non‐frail	Frail		
	M (SD)	M (SD)	T‐test results^a^	p value	M (SD)	M (SD)	T‐test results^a^	p value
Number of teeth	20.02 (6.92)	18.20 (7.53)	t (101.31) = 1.52	.131	20.13 (6.86)	17.23 (6.95)	t (80.53) = 2.51	**.014**
Number occlusal contacts	7.76 (2.75)	7.27 (3.46)	t (94.84) = 0.95	.345	7.77 (2.80)	6.57 (3.67)	t (67.08) = 2.09	**.040**
Percentage occlusal contacts	84.46 (24.08)	73.76 (32.75)	t (73.00) = 2.08	**.041**	84.29 (24.40)	74.32 (30.37)	t (53.97) = 1.91	.062
DPSI	3.75 (1.16)	3.60 (1.31)	t (101,49) = 0.73	.470	3.73 (1.21)	3.65 (1.19)	t (80.19) = 0.41	.685
Plaque index	0.99 (0.74)	1.21 (0.75)	t (115.21) = −1.93	.056	1.00 (0.72)	1.37 (0.80)	t (75.39) = −2.86	**.006**

	SPQ							
	Non‐frail	Frail	t‐test results^a^	*p* value				
	M (SD)	M (SD)						
Number of teeth	20.33 (6.95)	18.25 (7.14)	t (185.58) = .2.02	**.045**				
Number occlusal contacts	8.12 (2.56)	6.90 (3.33)	t (183.83) = 2.90	**.004**				
Percentage occlusal contacts	82.11 (27.20)	81.65 (26.52)	t (179.29) = 0.12	.908				
DPSI	3.70 (1.18)	3.78 (1.23)	*t* (194.34) = −0.46	.647				
Plaque index	0.98 (0.75)	1.24 (0.80)	*t* (193.99) = −2.37	**.019**				

Abbreviation: DPSI, Dutch Periodontal Screening Index; SPQ, Sherbrooke Postal Questionnaire

^a^ Assuming unequal population variances

p values <.05 are printed in bold

### 3.3 Correlations between oral health variables and frailty scales

Table 3 shows the correlations between the four frailty scales (TFI, GFI, SC, SPQ) and the nine oral health variables. The SC was significantly correlated with four of the oral health variables: DMFT, number of teeth, percentage occlusal contact and Plaque Index. The TFI was correlated with number of teeth, percentage occlusal contact and Plaque Index. The SPQ was correlated with the number of teeth and the number of occlusal contacts, respectively. Finally, the GFI was only correlated with DPSI. Number/percentage of decayed teeth and the Augsburger Index were not correlated with any of the frailty scales.

**TABLE 3:** Correlations between oral health variables and frailty scales

	TFI		GFI		SC		SPQ	
	*r*	p value	*r*	p value	*r*	p value	*r*	p value
DMFT	0.145	.052	0.083	.248	0.174	**.014**	0.123	.083
Number of teeth	−0.168	**.028**	−0.127	.083	−0.178	.**015**	−0.173	.**018**
Number decayed teeth	0.072	.338	0.023	.752	0.050	.486	0.012	.864
Percentage decayed teeth	0.053	.476	0.025	.727	0.024	.735	0.019	.789
Number occlusal contacts	−0.143	.056	−0.137	.055	−0.137	.052	−0.243	**<.001**
Percentage occlusal contacts	−0.156	**.046**	−0.056	.454	−0.187	**.011**	−0.073	.326
DPSI	−0.126	.093	−0.168	**.019**	−0.025	.729	−0.050	.488
Plaque index	0.187	**.013**	0.118	.101	0.226	**.001**	0.136	.058
Augsburger index	0.054	.645	0.019	.869	0.033	.768	‐0.074	.504

Abbreviation: GFI, Groningen Frailty Indicator; SC, Sunfrail Checklist; SPQ, Sherbrooke Postal Questionnaire; TFI, Tilburg Frailty Indicator

p values <.05 are printed in bold

## 4 DISCUSSION

The aim of this pilot study was to determine the associations between four validated multidimensional self‐report frailty scales (TFI,^4^ GFI,^5^ SC,^6^ SPQ^7^), and nine indices of oral health, with the aim of identifying and anticipating on poor oral health and frailty at an early stage. In a way that preventive care can be used to prevent deterioration of oral health and ensure maintenance of quality of life. Furthermore, early detection of frailty by oral care professionals could contribute to interprofessional management of frailty. Although the multidimensional scales TFI, GFI, SC, and SPQ all contain physical, psychological, and social frailty items, the findings are different. People declared to be frail according to the SC and SPQ had a lower number of teeth, less occlusal contacts and more plaque compared to non‐frail people. In addition, those declared to be frail according to the TFI had a significantly lower percentage occlusal contacts compared with non‐frail people.

Previous studies examined associations between oral health and physical frailty,^12^, ^31^ mostly defined according to the physical phenotype of frailty by Fried et al.^2^ As described in the introduction section a recently published systematic review on the relationship between oral health factors and frailty (unidimensional and multidimensional) resulted in twelve oral health factors, which were grouped in four different categories. The four categories were: (1) Factors of oral health status deterioration (52%), in particular few remaining teeth (29%); (2) Reduced oral motor skills (27%), especially masticatory function (9%), oral diadochokinesis (5%), occlusal force (7%); (3) chewing, swallowing, and saliva disorders (20%), especially chewing difficulties (11%); and (4) oral pain (1%).^15^

Unfortunately, European dentists are not trained to assess decline in reduced oral motor skills or chewing and swallowing function at this moment. Within their scope, oral health status deterioration (measured by DMFT and Plaque index) and chewing function (measured by number of remaining teeth and occlusal contacts) are the best predictors for frailty. Unlike in epidemiological research, in general practice recent caries activity can be monitored. This is a better predictor for frailty than DMFT, as the latest is an accumulation over life. By monitoring these factors, dentists can play a role in the early detection of frailty and anticipate for the maintenance of oral health. With regard to the interprofessional management of frailty, the SC and TFI might help to identify older people with risk of poor oral health and overall frailty so that an interprofessional care plan can be created for them to prevent deterioration, incidents and hospitalization.

The findings that oral health variables (DMFT, number of teeth, number/percentage occlusal contacts, Plaque Index) were correlated with frailty are partly supported by previous studies.^32^, ^33^, ^34^ Among 4720 older people ≥65 years included in the Obu Study of Health Promotion for the Elderly, it was shown that fewer present teeth was associated with frailty when assessed with the phenotype of frailty.^34^ Moreover, non‐denture users with <20 teeth showed higher odds for musculoskeletal frailty, determined by handgrip strength (OR 1.32, 95% CI 1.04–1.68).^33^ It is not surprising that the Plaque Index was correlated with frailty as oral hygiene habits are related to frailty.^13^, ^35^, ^36^ The Augsburger Index showed no significant correlations with the four frailty scales which can be explained by the small sample size of denture‐wearing participants within the study. Additionally, for frail older people denture hygiene might be easier to achieve than oral hygiene. In contrast with the findings from a longitudinal epidemiological cohort study in the urban area of Sydney (Australia), no associations were found between decayed teeth and frailty.^37^ However, this study did not find an association between frailty and DPSI; in the present study DPSI was correlated with frailty assessed with the GFI. This is possibly due to the difference in the designs, as the Australian study was longitudinal, while ours was cross‐sectional.

Some limitations of the present study should be mentioned. First, a selection bias may have been introduced by asking participants to participate during their dental visit. Most likely participants differ in socio‐economic and frailty characteristics from those who do not visit their dentist. This is important, as the ability to visit the dental practice is related to the degree of (physical) frailty. Prevalence of frailty varied from 24.5% (SC) to 49.7% (SPQ). Indeed, these figures are lower than in similar studies conducted in samples of community‐dwelling people, for instance 28.4% in a sample of 195 Dutch people (mean age 77.4; standard deviation 5.1) using the SC^24^ and 59.1% in a sample of 532 Dutch people (mean age 77.2; standard deviation 5.5) using the SPQ.^38^ It is also likely that their oral health is better than those with lower levels of dental service utilization. Therefore, it is assumed that the reported oral health status is better compared to the older Dutch population in general; unfortunately, these data were not available. Second, the use of two dental examiners may have led to different interpretations and scores in spite of their calibration. An examination of the inter‐examiner reliability did not occur. Third, the selection of the two dental practices in different areas of the Netherlands may have introduced bias. However, it may also contribute to a better generalization of the data to the entire Dutch population aged 70 years or older. Fourth, all the data with regard to frailty and chronic diseases were based on self‐report. Answering questions about frailty in a dental practice can result in socially desirable responses. However, a study using data from The Irish Longitudinal Study on Ageing (TILDA) showed that, except for gender differences, characteristics of frailty are similar regardless of whether self‐report scales or physical tests are used.^39^ Finally, in terms of age, there was an underrepresentation in the 70 to 74 age group and an overrepresentation in the 75 to 79 age group relative to the number of patients enrolled at the dental practices, caused by a decision to reduce the age of the participants in order to reach the desired number of 200 participants sooner. In terms of gender, however, the participants were representative of the patient groups. A strength of the present study is also the response rate; only 17 people decided not to participate in the scientific research, providing a response rate of 92.4%.

Because standard oral health variables alone often do not correlate with frailty, we recommend that dental care providers collect more information about the general health and frailty status of their older patients, and whether they visit other (dental) healthcare professionals. Ideally, dental care providers should inform the general practitioner about their concerns in order to lead to early interventions. Similarly, when a general practitioner detects frailty there is a possibility that the oral health of this patient will decline and a recommendation to visit a dental practice is necessary. This asks for awareness and behavioral changes in both professions to enhance interdisciplinary collaboration in primary care services for older patients at neighborhood level. In future, we have to respond to the problem more in a multidisciplinary approach at a local level. The use of multidimensional frailty scales is strongly recommended. Setting up a multidisciplinary department at universities will give an incentive to this need.

In conclusion, the present study showed that several self‐report multidimensional frailty scales each correlated differently with oral health variables. Of the studied multidimensional frailty scales, the SC and the TFI seem to be most appropriate for identifying older people with risk of poor oral health, because they were correlated with the most oral health variables (four and three, respectively). However, it should be noticed that even these correlations were small. Further research aiming to determine the associations between multidimensional frailty and oral health in a large sample and a longitudinal design is recommended.

## AUTHOR CONTRIBUTIONS

Erik M. van der Heijden, Wim J. Kluter, Claar D. van der Maarel–Wierink, and Robbert J J. Gobbens contributed to conceptualization and design of the study, drafting the article and approved the final version of the article. Erik M. van der Heijden and Wim J. Kluter were responsible for collecting the data. Wim J. Kluter and Robbert J J. Gobbens conducted the statistical analyses and were responsible for interpretation of the data. Robbert J J. Gobbens supervised the study.
